# Biomimetic Coating Technologies and Biomaterials for Medical Applications

**DOI:** 10.3390/biomimetics11100734

**Published:** 2026-10-14

**Authors:** Valentina Grumezescu, Liviu Duta

**Affiliations:** National Institute for Laser, Plasma and Radiation Physics, 077125 Magurele, Romania; valentina.grumezescu@inflpr.ro

Current advancements in medical technologies continue to push the boundaries of what is possible in terms of patient care and treatment outcomes. One of the most exciting and promising approaches in this domain is the development of various biomaterials and biomimetic coating technologies. These innovations aim to emulate the remarkable properties of natural biological systems, thereby enhancing the functionality and compatibility of implants and medical devices.

Biomimetic coatings and biomaterials are designed to interact harmoniously with biological tissues, promoting integration and reducing adverse reactions. Inspired by nature’s own solutions, these materials leverage the intricate structures and functions found in living organisms. The applications of biomimetic coatings and biomaterials are vast and varied, encompassing areas such as orthopedics, cardiology, dentistry, and wound healing. For example, in orthopedics, biomimetic coatings and surface functionalization strategies can enhance implant osseointegration by promoting osteoblast activity and bone formation at the implant surface [1]. In cardiology, bio-inspired and biomimetic surface modifications can be applied to cardiovascular stents to reduce thrombosis and restenosis by promoting endothelialization, inhibiting platelet activation, and limiting smooth muscle cell proliferation [2,3]. Meanwhile, in the realm of wound healing, biomimetic materials can promote endogenous skin regeneration and may help reduce scar formation [4]. Beyond these applications, recent developments in bioactive biomaterial interfaces, controlled drug-delivery systems, biodegradable and bioresorbable scaffolds, extracellular-matrix-inspired materials, and vascularized tissue-engineering constructs illustrate the broad and increasingly interdisciplinary scope of biomimetic strategies in regenerative medicine and tissue engineering [5,6,7]. In particular, advances in bioceramic scaffolds, 3D-printed composites, and hydroxyapatite–hydrogel systems offer promising strategies for bone and osteochondral tissue regeneration [8], while tailoring the electrical and dielectric properties of hydroxyapatite-based materials through doping represents an additional approach to improving their functionality for biomedical applications [9].

Against this background, the contributions collected in this Special Issue (titled Contribution 1 to Contribution 10), entitled “Biomimetic Coating Technologies and Biomaterials for Medical Applications”, illustrate the diversity of current approaches to designing biologically inspired surfaces and materials, ranging from inorganic and polymeric coatings for metallic implants to collagen-based interfaces, electroactive materials, drug-loaded fibrous systems, and advanced strategies for regenerative medicine. Together, the ten contributions highlight how surface chemistry, micro- and nanotopography, biomolecular functionalization, controlled therapeutic delivery, and the recreation of native tissue architectures can be exploited to improve interactions between engineered materials and biological environments.

The contributions included in this Special Issue can be broadly organized into three interconnected thematic areas, reflecting complementary approaches to the development and application of biomimetic coatings and biomaterials in medicine.

(i) Biomimetic Coatings and Surface Engineering for Implantable Materials

Cotrut et al. (2024) [Contribution 1] investigated the deposition of biomimetic hydroxyapatite (HAp) coatings on titanium (Ti) substrates utilizing a pulsed galvanostatic method. In vitro cytocompatibility evaluations conducted with human mesenchymal stem cells (hMSCs) revealed that the HAp-coated Ti samples supported high cell viability, type I collagen synthesis, localized fibronectin deposition, and increased alkaline phosphatase activity, demonstrating biocompatibility and early osteogenic differentiation. Moreover, biomineralization and electrochemical investigations demonstrated the beneficial effect of the HAp coatings on the properties of the Ti substrate, including enhanced corrosion resistance and apatite-forming ability in simulated body fluid. The developed coatings consisted of thin, wide, plate-like crystals oriented perpendicular to the Ti substrate, exhibited a Ca/P ratio close to that of stoichiometric HAp, and presented a hydrophilic character [Contribution 1].

A natural deep eutectic solvent (NADES)-based electropolymerization strategy was developed to deposit polypyrrole (PPy) and Naproxen-doped PPy (NAP-PPy) films onto a biomedical high-entropy alloy in the study reported by Nartita et al. (2025) [Contribution 2]. These multifunctional PPy–NAP films combine robust corrosion protection with sustained and controlled Naproxen release, supporting their potential biomimetic role as smart coatings for next-generation implantable devices. Electrochemical tests in simulated body fluid indicated increased corrosion protection provided by both PPy and PPy–NAP films. Compared to the pristine substrate’s corrosion current density of 754 µA/cm^2^, PPy reduced this value to ~4.5–7.0 µA/cm^2^, while PPy–NAP reduced it to ~1.3–5.5 µA/cm^2^. Naproxen incorporation also enabled sustained drug release, reaching ~94–98% after eight days, depending on the deposition mode. PPy–NAP films on Ti20Zr5Ta2Ag therefore combine improved electrochemical stability with controlled drug-release behavior and enhanced surface wettability. These attributes suggest their suitability for further investigation as multifunctional coatings in biomedical implant applications [Contribution 2].

Complementing these experimental approaches, the systematic literature analysis performed by Min and co-workers (2026) [Contribution 3] underlined the profound potential of biomimetic coacervates as a cornerstone platform for next-generation multifunctional medical devices and personalized regenerative medicine. This work outlines recent advancements in biomimetic coacervate coatings, detailing their development from fundamental physical–chemical principles of liquid–liquid phase separation (LLPS) to advanced biomedical applications. The authors summarized recent developments published between 2020 and 2025 in multifunctional biomedical applications, including wet tissue sealing, bone repair, localized stimuli-responsive drug or nucleic acid delivery, anti-biofouling medical device coatings, and regenerative cell–material interfaces. The review further emphasized that future efforts should focus on establishing adaptable quality-control frameworks based on system-specific analytical benchmarks. In particular, industrial scaling through continuous-flow technologies will require in-line physical monitoring, standardized rheological profiling, and substrate-tailored mechanical benchmarks under simulated physiological environments.

Negrescu and co-workers (2025) [Contribution 4] fabricated two different types of anodic TiO_2_ nanotubes, namely TNTs with only minor spacing at the tube top and spTNTs, characterized by distinct tube-to-tube spacing. By tailoring the anodization parameters, nanotubes with similar morphological parameters in terms of tube diameter, spacing, and tube length were obtained on different Ti substrates (i.e., foils and pins), allowing previous in vitro observations to be further investigated under in vivo conditions [Contribution 4]. The layers were evaluated regarding their potential to promote new bone formation and thereby improve the osseointegration process at the implantation site. Although the two nanotubular morphologies exhibited similar chemical composition and comparable hardness and elastic modulus, the spaced nanotubes showed greater surface roughness. Importantly, histological evaluation at one month after implantation demonstrated that the spTNT-coated pins promoted significantly greater early new bone formation, as reflected by a higher mean thickness of newly formed bone tissue, than the TNT-coated implants, emphasizing the relevance of nanotube lateral spacing in the design of dental and orthopedic implant surfaces [Contribution 4].

Manescu (Paltanea) et al. (2026) [Contribution 5] provided a comprehensive analysis of the current options and future perspectives for conversion coatings applied to biodegradable Mg-based alloys. These alloys are promising materials for temporary implants because of their biodegradability and elastic modulus close to that of bone; however, their rapid corrosion in physiological environments remains a major limitation to clinical translation. The review discusses ceramic, polymeric, bioactive, and hybrid conversion coatings as strategies for controlling the degradation rate while simultaneously improving surface biocompatibility. Particular attention is devoted to the relationship between corrosion behavior and biological performance, including the osteogenic and angiogenic signaling pathways associated with different coating systems. The authors emphasize that, despite significant progress, achieving an appropriate balance between Mg-alloy degradation and new bone formation remains a central challenge. Future developments should therefore focus on improved control of coating composition, microstructure, degradation behavior, and cellular responses, together with the development of hybrid and stimuli-responsive systems capable of providing both corrosion protection and bioactive functionality [Contribution 5].

(ii) Biofunctional Polymers, Collagen-Based Materials, and Biomimetic Interfaces

Another study regarding surface biofunctionalization described a method for the surface functionalization of polyvinylidene fluoride (PVDF) films with biological molecules, as reported by Leiva et al. (2025) [Contribution 6]. To exploit the full potential of PVDF in biomedical applications and, specifically, for the creation of biomimetic piezoelectric implants with improved cellular affinity, it is essential to overcome the limitations associated with its synthetic nature and inherent hydrophobicity. The developed method provided comparable collagen densities independently of the surface potential of the PVDF film while largely retaining its piezoelectric properties. This approach may also facilitate investigations of the transduction pathways involved in cellular responses to piezoelectric signals. By covalently immobilizing type I collagen onto β-phase PVDF membranes, the authors developed a biomimetic composite that combines two relevant features of natural bone. This construct leverages the piezoelectric properties of PVDF to mimic the electroactive characteristics of bone while simultaneously offering a collagen-functionalized surface for enhanced cell attachment. Consequently, this functionalization strategy addresses important biomedical limitations of PVDF, improving its biocompatibility and expanding its potential for advanced tissue-engineering applications, including bone-related ones [Contribution 6]. The biological rationale is particularly relevant because piezoelectric scaffolds can reproduce electroactive characteristics of native tissues and have the potential to promote the integration of implanted materials with host tissues.

Nashchekina and Nashchekin (2025) [Contribution 7] comprehensively delineated the methods and conditions for obtaining oriented collagen fibrils, which can help in the development of tissue biomimetic materials with complex properties resembling those of native tissues. The authors summarized various methods and conditions for obtaining oriented collagen fibrils in vitro using mechanical, electric, magnetic, and other fields. The prospects for their application in tissue engineering and the scientific challenges associated with oriented collagen fibrils are also described. The review emphasizes that fibril orientation represents an important structural feature of native tissues such as bone, cartilage, tendons, and the cornea and can influence cell migration and extracellular matrix organization. However, despite the diversity of available orientation approaches, reproducing native-like collagen organization in vitro remains challenging, highlighting the need for a deeper understanding of the mechanisms governing collagen fibril orientation and assembly [Contribution 7].

Another detailed overview of recent progress in collagen-based biomaterials was provided by Grumezescu and Duta (2026) [Contribution 8], who focused on collagen type I as a biological barrier interface in biomimetic microfluidic devices. The authors highlighted the ability of its fibrillar architecture, native cell-binding ligands, and susceptibility to cell-mediated remodeling to support epithelial and endothelial polarization, tight-junction formation, and physiologically relevant transport behavior. Recent technological developments include ultrathin collagen barriers for improved molecular exchange, gentle crosslinking, and composite approaches to limit compaction and delamination under flow, and patterning or bioprinting strategies that allow control over alignment and graded porosity and facilitate integration into microfluidic geometries. Applications encompass models of the intestine, vasculature, skin, airway, kidney, and tumor–stroma interfaces. At the same time, challenges related to batch variability, long-term mechanical drift, standardization of fibrillogenesis conditions, and scale-up without loss of bioactivity remain to be addressed. The review therefore positions collagen type I as an important biomimetic interface for developing increasingly physiologically relevant microfluidic and organ-on-chip platforms [Contribution 8].

(iii) Bio-inspired Platforms for Therapeutic Delivery and Regenerative Medicine

Novel electrospun polymer mats based on biocompatible poly(lactic acid) (PLA) and hydrophilic poly(ethylene glycol) (PEG) were successfully fabricated for the co-delivery of two natural polyphenols, quercetin (QUE) and rosmarinic acid (RA), as reported by Stoyanova et al. (2026) [Contribution 9]. In this study, the authors fabricated and characterized PLA/PEG electrospun fibers co-loaded with QUE and RA, evaluated the antioxidant activity and mechanical performance of the fabricated fibrous materials, and investigated the biological response of HeLa cervical carcinoma cells and normal BALB/3T3 fibroblasts following exposure to the developed materials. The resulting defect-free nanofibrous structures exhibited high interconnected porosity and mimicked structural characteristics of the native extracellular matrix. The PLA/PEG/QUE/RA mats exhibited the highest antioxidant efficiency (98.1%), while the biological investigations demonstrated pronounced anticancer activity against HeLa cells, with normal BALB/3T3 fibroblasts being comparatively more resistant, suggesting a degree of selectivity toward cancer cells. The results obtained suggest that these novel materials have potential for further development for applications in localized cancer therapy, wound management, and tissue engineering.

Ultimately, the review published by Kawai (2025) [Contribution 10] underscores that successful bone tissue engineering relies heavily on vascularization to maintain graft viability and prevent central necrosis caused by insufficient nutrient supply and waste removal. Recent years have witnessed substantial progress in engineering vascularized networks within bone substitutes. The strategies discussed include three-dimensional (3D) bioprinting approaches, biomaterial scaffolds containing microchannels and high-surface-area architectures, biochemical interventions, and cell co-culture techniques. Advanced 3D-bioprinting approaches, including inkjet, extrusion, and laser-assisted methods, enable the spatial organization of endothelial and osteogenic cells, while the combined delivery of angiogenic and osteogenic factors, such as VEGF and BMP-2, can promote the coupling between angiogenesis and osteogenesis. Co-culture approaches involving mesenchymal stem cells and endothelial progenitors further support the development of functional capillary networks. Preclinical studies have demonstrated enhanced vascular inosculation, increased bone formation, and improved mechanical stability in pre-vascularized grafts compared with non-vascularized controls. While clinical translation and the achievement of stable and functionally mature vascular networks remain important challenges, these multifaceted strategies have substantially enhanced graft viability and regenerative performance, reinforcing vascularization as a fundamental pillar of successful bone tissue engineering [Contribution 10].

Taken together, the contributions to this Special Issue demonstrate the breadth of contemporary biomimetic strategies for medical applications and the increasingly close integration of surface engineering, biomaterials science, cell biology, and regenerative medicine. The studies addressing implant surfaces illustrate how electrochemically deposited HAp, drug-loaded conductive polymers, biomimetic coacervates, TiO_2_ nanotopographies, and conversion coatings for biodegradable Mg alloys can be tailored to regulate corrosion, bioactivity, therapeutic delivery, cellular responses, and osseointegration. In parallel, the investigations of collagen-functionalized piezoelectric PVDF and oriented collagen fibrils emphasize the importance of reproducing structural, biochemical, and electroactive characteristics of native extracellular matrices, while collagen-based microfluidic barrier interfaces extend these concepts toward increasingly physiologically relevant in vitro models. Finally, electrospun PLA/PEG systems for the delivery of bioactive polyphenols and emerging strategies for vascularized bone tissue engineering demonstrate how biomimetic architectures can simultaneously provide structural support and biological functionality.

Collectively, these contributions underline a transition from biomaterials designed primarily for passive compatibility toward multifunctional systems capable of actively interacting with the biological environment. Continued progress in controlling material architecture, surface chemistry, therapeutic delivery, degradation behavior, and cell–material interactions, together with improved standardization and clinically relevant validation, will be essential for translating these biomimetic concepts into next-generation medical devices, implants, and regenerative therapies.

## Data Availability

No new data were created or analyzed in this study. Data sharing is not applicable to this article.

## References

[1] Stewart C., Akhavan B., Wise S.G., Bilek M.M.M. (2019). A review of biomimetic surface functionalization for bone-integrating orthopedic implants: Mechanisms, current approaches, and future directions. Prog. Mater. Sci..

[2] Rao J., Bei H.P., Yang Y., Liu Y., Lin H., Zhao X. (2020). Nitric oxide-producing cardiovascular stent coatings for prevention of thrombosis and restenosis. Front. Bioeng. Biotechnol..

[3] Weng Y., Chen J., Tu Q., Li Q., Maitz M.F., Huang N. (2012). Biomimetic modification of metallic cardiovascular biomaterials: From function mimicking to endothelialization in vivo. Interface Focus.

[4] Wang M., Hong Y., Fu X., Sun X. (2024). Advances and applications of biomimetic biomaterials for endogenous skin regeneration. Bioact. Mater..

[5] Vahidi M., Rizkalla A.S., Mequanint K. (2024). Extracellular matrix-surrogate advanced functional composite biomaterials for tissue repair and regeneration. Adv. Healthc. Mater..

[6] van Kampen K.A., Mota C., Moroni L. (2025). A tissue engineering’s guide to biomimicry. Macromol. Biosci..

[7] Aarya Sree K., Yagnik A., Chordiya P.K., Choudhury K., Kumar P. (2023). Nature-inspired vascularised materials and devices for biomedical engineering. Curr. Opin. Biomed. Eng..

[8] Visan A.I., Duta L., Negut I. (2026). Innovative Hydroxyapatite–Hydrogel Composites for Cartilage Regeneration. Gels.

[9] Duta L., Grumezescu V. (2024). The Effect of Doping on the Electrical and Dielectric Properties of Hydroxyapatite for Medical Ap-plications: From Powders to Thin Films. Materials.

